# The Effect of a Hyperdynamic Circulation on Tissue Doppler Values: A Simulation in Young Adults during Exercise

**DOI:** 10.1155/2011/165874

**Published:** 2011-02-17

**Authors:** Colin F. Royse, Ni Ruizhi, Andrew L. Huynh, Alistair G. Royse

**Affiliations:** ^1^Department of Pharmacology, University of Melbourne, Level 8, Medical Building, 3010 Carlton, Australia; ^2^Department of Anaesthesia and Pain Management, The Royal Melbourne Hospital, 3050 Melbourne, Australia; ^3^Department of Cardiology, The First Affiliated Hospital of Kunming Medical University, Yunnan Province 650034, China; ^4^Department of Cardiothoracic Surgery, The Royal Melbourne Hospital, 3050 Melbourne, Australia

## Abstract

Left ventricular tissue Doppler imaging (TDI) velocities are used to monitor systolic and diastolic function, but it is not known how these may change in a hyperdynamic circulation, as often occurs in anesthesia and critical care medicine. Twenty-six healthy young volunteers were recruited and left ventricular systolic and diastolic tissue Doppler velocities measured at rest, light exercise, strenuous exercise, and recovery (10 minutes after exercise). At rest, TDI velocities significantly decreased from base to apex (*P* < .001). Within basal, mid, and apical sections, systolic and diastolic peak velocities differed between segments (*P* < .05), except for systolic middle (*P* = .094) and late diastolic apical velocities (*P* = .257). Basal septal velocities differed from basal lateral, for systolic (*P* = .041) but not diastolic peak values. Inferobasal radial values differed from basal lateral values for both systolic and diastolic velocities (*P* < .05). Both systolic and diastolic TDI velocities increased significantly in all segments in a proportionate manner with a hyperdynamic circulation.

## 1. Introduction

Tissue Doppler imaging is commonly used to assess diastolic function, and for the assessment of left atrial pressure. These measurements are most commonly applied to outpatients under resting conditions. The normal values reported in outpatients may not apply during acute changes in hemodynamic state, which occurs frequently in intensive care patients or those undergoing anesthesia. Assessment of left ventricular systolic and diastolic function during exercise using tissue Doppler imaging (TDI) has been used to detect underlying pathology, such as ischemic heart disease [1].

It is important to determine whether left ventricular systolic and diastolic tissue Doppler velocity values are similar in all segments in young healthy adults in order to identify if values from different segments can be used interchangeably. Exercise induces a hyperdynamic circulation, and TDI values may alter from resting conditions. Such changes assessed in normal young adults may be indicative of changes that should be expected in pathological hyperdynamic circulation states, with the premise that an absence of predicted change could indicate pathology. 

The aim of the study was to assess changes in left ventricular systolic and diastolic tissue Doppler velocities during a hyperdynamic circulation, in normal healthy subjects under resting and exercise conditions.

## 2. Materials and Methods

### 2.1. Subjects

Twenty-six healthy volunteers, between the ages of 18 to 22 years, were recruited. Written, informed consent was obtained. This study was approved by The University of Melbourne Human Research Ethics Committee in accordance with the Code of Practice of the National Health and Medical Research Council of Australia. 

The study was conducted in the Human Laboratory of the Cardiovascular Therapeutics Unit, Department of Pharmacology, University of Melbourne.

### 2.2. Echocardiography

All recordings were performed with a SonoSiteMicroMaxx (Sonosite Australia, NSW, Australia) echocardiography machine with a 2.5 MHz phased array transducer. A comprehensive transthoracic study was first performed to exclude any unexpected pathology. Subjects were in the lateral decubitus position and simultaneously monitored with an electrocardiogram (ECG). 

Echocardiography images were digitally recorded and analysed offline using Prosolv Cardiovascular Analyser (Vision Software, QLD, Australia). Each measurement was the average of 3 consecutive beats from 2 observers.

Demographic data, heart rate, and blood pressure were recorded prior to echocardiography. The heart segments were imaged from apical views; the radial TDI from the parasternal short axis view, and M-Mode assessment of left ventricular function, was performed from the parasternal long axis view. 

Recordings were made at the end of each condition. Exercise recordings were measured at apical basal septal and lateral views and parasternal short axis inferobasal view. 

For postexercise recordings TDI recordings were performed for basal septal and basal lateral segments and the inferobasal (radial) segment, in order to minimise the risk of changes in circulatory parameters during the period of recording.

### 2.3. Exercise Protocol

Exercise was performed by running on a treadmill. 

Recordings were performed at 4 time periods.

- Baseline—resting phase.
- Low-intensity exercise—running on a treadmill for 2 minutes or until reaching a heart rate of 100–120 beats·min^−1^.
- Heavy-level exercise—running on a treadmill for 5 minutes or until reaching a heart rate of greater than 140 beats·min^−1^.
- Recovery—10 minutes after heavy exercise.

Heart rate and blood pressure were monitored with each condition.

### 2.4. Statistics

Data are presented as mean ± standard deviation. Values from baseline to exercise were analysed by repeated measures ANOVA for within-subject difference with Greenhouse-Geisser correction for multisampleasphericity. Statistical significance was defined as *P* < .05. Graphs were constructed using GraphPad Prism 5.0., and analyses were performed using SPSS version 14.0.

Interobserver variability analysis was done using the Bland and Altman 95% limits of agreement method [2]. The mean is the average of two observers. Mean difference is the difference of means between two observers expressed as a positive value. The “limits of agreement” was defined as two standard deviations of the difference and expressed as a percentage of the average value of the mean difference. An acceptable limit of agreement is ±30% [3].

## 3. Results

Twenty-nine volunteers were enrolled and 3 were withdrawn because of inadequate imaging or inability to complete the exercise protocol. Twenty-six subjects were included in the study. There were 11 males and 15 females, with a mean age of 19.8 ± 1.5 years (18–22), height of 169.7 ± 7.2 cm (157–186), and weight of 64.6 ± 10.2 kg (50–104). Hemodynamic variables during the exercise protocol are summarised in Table 1. All subjects were found to have a normal left ventricular mass (mean mass 119.4 g ± 6.8). Ejection fraction calculated using Teichholz formula was 53.5 ± 0.8%. 

### 3.1. Resting Values

**Figure 1:**
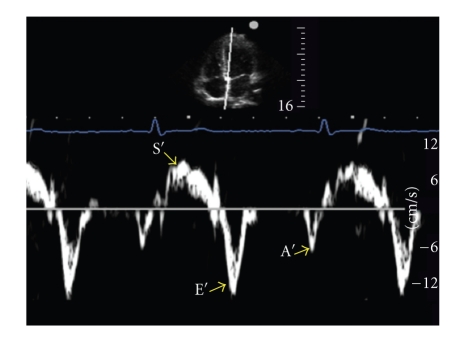
Spectral tissue Doppler tracing for the apical 4-chamber transthoracic window, showing the cursor position on the septal basal segment. The transducer is placed at the apex of the heart and angled towards the head.

Within each ventricular wall, velocities decreased significantly from basal to apical segments (*P* < .001) (Figure 1 and Table 2). There was no significant difference in radial TDI for the inferobasal or anterobasal segments.

**Figure 2:**
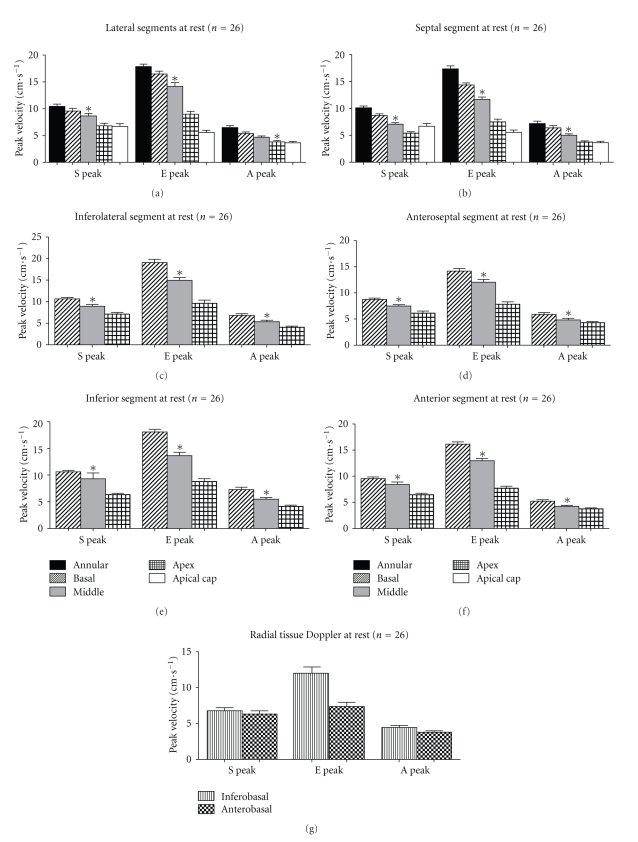
Resting Tissue Doppler velocities for each ventricular wall ((a)–(f)) and for radial measurements of the inferobasal and anterobasal segments (g). S: systolic velocity; E: early diastolic velocity; A: late diastolic velocity; Error bars are standard error of the mean. *P < .001. *P* values are determined for the comparison of values from basal to apical segments using repeated measures ANOVA for within subject difference with Greenhouse-Geisser correction.

Within the base, middle, and apical sections of the heart, velocities were significantly different between segments for both systolic and diastolic measurements (*P* < .05), with the exception of middle section systolic velocities (*P* = .094) and the apical late diastolic velocities (*P* = .257) (Figure 2). There was no difference in diastolic TDI velocities for septal basal and lateral basal segments, but there was a small but significant difference in systolic velocities (*P* = .041). For radial TDI measurements, the inferobasal segment velocities were significantly lower than the anterobasal segment (systolic (*P* < .001), early diastolic (*P* < .001), and late diastolic velocities (*P* = .009)).

### 3.2. Exercise Values

**Figure 3:**
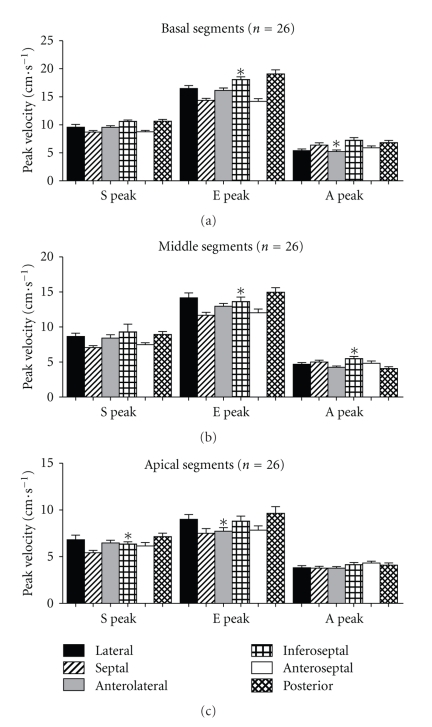
Resting tissue Doppler velocities for basal (a), middle (b), and apical (c) sections. S: systolic velocity; E: early diastolic velocity; A: late diastolic velocity; Error bars are standard error of the mean. *P < .001. *P* values are determined for the comparison of values from basal to apical segments using repeated measures ANOVA for within subject difference with Greenhouse-Geisser correction.

Tissue Doppler velocities changed significantly, proportionate to exercise effort, in all segments studied during the exercise and recovery (all comparisons *P* < .001) (Figure 3 and Table 3).

### 3.3. Interobservor Variability

Interobservor variability data for echocardiography measurements are summarised in Table 4. All variables were within accepted limits of agreement of ±30% [3].

## 4. Discussion

**Figure 4:**
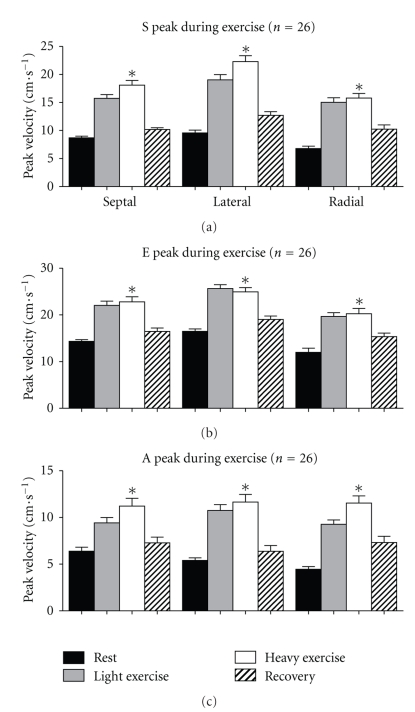
Tissue Doppler values during exercise for systolic (a), early diastolic (b), and late diastolic velocities (c). Septal is septal basal segment, lateral is the lateral basal segment, and radial is the inferobasal segment measured in the short axis. *P < .001. *P* values are determined for the comparison of values from basal to apical segments using repeated measures ANOVA for within subject difference with Greenhouse-Geisser correction.

Our study showed that tissue Doppler velocities are not uniform across different myocardial segments, either within wall (base to apex) or between basal, middle, or apical sections. The highest systolic and diastolic velocities occur in the basal segments and decrease significantly from base to apex. During exercise all tissue Doppler values increased proportionate to effort and reduced with recovery (Figure 4). 

Our results may have implications for critically ill patients, particularly those with hyperdynamic circulations. Apical views are at times difficult to acquire in supine ventilated patients; images that can be obtained may be foreshortened, or more apically placed segments may be the only segments visible. Values between segments differ and should not be considered equivalent when diagnosing systolic or diastolic abnormality. Secondly, our data shows that values *should increase* in a hyperdynamic circulation, indicating that even “normal” values may represent pathology during hyperdynamic circulation conditions. However, we urge caution in directly translating these findings to the critically ill, where the disease process may produce very different haemodynamic conditions to the exercising young adult.

The most commonly used segments in clinical practice are the septal basal and lateral basal segments. The diastolic velocities were equivalent, and thought the systolic values differed, the difference was small and not clinically important. This may not be the case, however, when there is regional disease in the basal myocardium. Cardim et al. [4], however, found that tissue Doppler velocities were higher in the lateral and inferior basal segments than in the septal and lateral segments for both systolic and diastolic function. The systolic and diastolic velocities in our study were higher than those reported in [4–6]. This could be due to the younger age of our cohort, and TDI values have been reported to decrease with age [6]. Lateral wall diastolic TDI values are reported to be higher than septal velocities, which was consistent with the trend observed in this study.

There was a significant difference between the velocities in segments within the base, middle, and apex of the heart wall with exception to the systolic middle and late diastolic apical velocities. Therefore, using velocities interchangeably between segments is not possible. Greaves and Alam found similar results [5, 7], reporting that the septal segment velocities were lower than the anterior, lateral, and inferior sites and attributed this to “the presence of ample longitudinally orientated fibres in these parts of the left ventricle.” Lateral systolic and diastolic velocities were found to be greater than septal systolic and diastolic velocities. Therefore the ventricular walls have a physiological heterogeneity and the velocities cannot be used interchangeably. Cardim described that the difference between the two segments occurred due to the “sandwich” position of the septum in between ventricles, the septum having a greater physiological stress than the lateral segments, the septalcytoarchitecture being rich in circumferential fibres, the degrees of interstitial fibrosis, and the proportion of myocyte/adrenergic receptors in comparison to the lateral wall [4]. Our study found significant differences between the lateral and inferior segments for both systolic and diastolic velocities. Radial values were lower than longitudinal values. It is therefore not possible to use radial tissue Doppler velocities as a substitute for longitudinal velocities. Greaves found radial values were lower than longitudinal [5].

During exercise, values for systolic and diastolic tissue Doppler imaging measurements increased proportionate to effort and reduced during recovery. There was a significant increase in all velocities in all segments at different degrees of exercise. Wilkenshoff looked at regional systolic velocity in 20 normal subjects during bicycle ergometry and found the velocities to increase during exercise [8]. 

Studies on left ventricular response during exercise have shown an increase in left ventricular ejection fraction [9–11]. There is an early increase in the ejection fraction and a tendency to decrease the ejection fraction as peak exercise is approached. The changes in tissue Doppler velocities in our studies reflect a similar effect, though the subjects were not exercised to maximal effort.

Stress echocardiography can be useful in investigating ischaemia and help increase our understanding of the physiological effects of ischaemia. Tissue Doppler imaging gives a quantitative and regional measure and is beneficial in measuring these changes. Exercise induced abnormalities of left ventricular function have high sensitivity and specificity for ischaemia (>90%) [10]. A decrease in regional wall motion and left ventricular ejection fraction is considered a diagnostic hallmark of exercise induced myocardial ischaemia [10]. This indicates the location of an abnormality. An increase in TDI velocities with exercise is an expected response, and it is possible that an absence of increase, or decreased velocities could indicate regional pathology. More research in patients with known ischaemic disease is required to answer this question.

The primary limitation is that we used exercise to simulate a hyperdynamic circulation. Healthy young adults were enrolled so that systolic and diastolic function would not be affected by possible medical comorbidities. Apical velocities were difficult to measure. Our study found that signal quality was reduced in the apical images, which could have contributed to lower recorded velocities in the apex, as well as differences in fibre alignment, and the angle dependence of Doppler imaging, which is enhanced in the apical region [12]. Echocardiographic studies during exercise are difficult to interpret due to the greater expansion of the lungs and higher respiratory frequency during exercise causing a reduction in the acoustic windows in most individuals [10]. It is considered optimal to acquire images in subjects in an upright position during exercise, but this is difficult. In our study, we obtained images in the supine left decubitus position immediately after exercise. Bougault has shown that left ventricular systolic and diastolic tissue Doppler imaging velocities are reproducible during exercise [13]. Further research is required in a sick cohort of patients to evaluate the impact of our findings.

## 5. Conclusion

Tissue Doppler velocities decrease from base to apex. There was a difference in velocities within the basal, middle, and apical segments. During hyperdynamic circulation velocities increase proportionate to effort and reduce with recovery.

## Figures and Tables

**Table 1:** Subject demographics and hemodynamic variables.

	Rest	Light exercise	Heavy exercise	Recovery	*P*
Heart rate	69.8 ± 10.5	120.0 ± 9.9	141.0 ± 13.0	93.3 ± 9.5	<.001
Systolic blood pressure (mm Mg)	123.4 ± 14.7	157.0 ± 18.8	146.4 ± 19.8	116.4 ± 8.4	<.001
Diastolic blood pressure (mm Hg)	66.5 ± 9.3	68.7 ± 11.7	68.3 ± 10.9	62.3 ± 10.2	.043

Values are mean ± standard deviation; *P* values are determined using repeated measures ANOVA for within subject difference with Greenhouse-Geisser correction for the comparison of values from baseline to recovery.

**Table 2:** Resting tissue Doppler velocities of segments.

Segment	Annulus	Basal	Middle	Apical	Apical cap	*P*
*Lateral*						
S′	10.4 ± 0.4	9.6 ± 0.5	8.7 ± 0.5	6.5 ± 0.3	6.7 ± 0.5	.002
E′	17.9 ± 0.4	16.5 ± 0.5	14.2 ± 0.7	8.7 ± 0.5	5.6 ± 0.4	<.001
A′	6.5 ± 0.4	5.4 ± 0.3	4.7 ± 0.2	3.8 ± 0.2	3.6 ± 0.3	<.001
*Septal*						
S′		8.7 ± 0.3	7.1 ± 0.3	5.4 ± 0.3		<.001
E′		14.4 ± 0.4	11.7 ± 0.4	7.5 ± 0.5		<.001
A′		6.4 ± 0.4	5.0 ± 0.3	3.8 ± 0.2		<.001
*Inferolateral*						
S′		8.8 ± 0.3	7.5 ± 0.4	6.1 ± 0.4		<.001
E′		14.2 ± 0.7	12.0 ± 0.6	7.8 ± 0.7		<.001
A′		5.9 ± 0.3	4.8 ± 0.4	4.3 ± .2		<.001
*Anteroseptal*						
S′		10.7 ± 0.2	8.9 ± 0.3	7.1 ± 0.4		<.001
E′		19.1 ± 0.5	15.0 ± 0.5	9.6 ± 0.4		<.001
A′		6.6 ± 0.3	5.4 ± 0.3	4.1 ± 0.2		<.001
*Anterior*						
S′		9.6 ± 0.3	8.1 ± 0.4	6.5 ± 0.3		<.001
E′		16.1 ± 0.4	13.0 ± 0.4	7.7 ± 0.4		<.001
A′		5.2 ± 0.3	4.2 ± 0.2	3.8 ± 0.2		<.001
*Inferior*						
S′		10.6 ± 0.3	9.3 ± 1.1	6.4 ± 0.2		<.001
E′		18.1 ± 0.5	13.6 ± 0.6	8.8 ± 0.5		<.001
A′		7.3 ± 0.5	5.5 ± 0.3	4.2 ± 0.2		<.001
*Inferobasal*						
S′		6.8 ± 0.4				
E′		12.0 ± 0.9				
A′		4.5 ± 0.3				
*Anterobasal*						
S′		6.3 ± 0.4				
E′		7.4 ± 0.6				
A′		3.8 ± 0.2				

Values are mean ± standard deviation. All velocities are in cm·sec^−1^; S′: peak systolic velocity; E′: peak early diastolic velocity; A′: peak late diastolic velocity. *P* values are determined using repeated measures ANOVA for within subject difference with Greenhouse-Geisser correction for the comparison of values from basal to apical segments.

**Table 3:** Changes in tissue Doppler velocities during exercise.

Segment	Baseline	Light exercise	Heavy exercise	Recovery	*P*
*Lateral*					
S′	9.6 ± 2.3	19.1 ± 4.6	25.8 ± 5.5	10.7 ± 2.7	<.001
E′	16.5 ± 2.4	22.3 ± 3.7	25.1 ± 4.7	11.7 ± 3.8	<.001
A′	5.4 ± 1.5	12.7 ± 2.9	19.0 ± 3.0	5.8 ± 2.7	<.001
*Septal*					
S′	8.7 ± 1.6	15.7 ± 3.5	22.0 ± 4.2	9.4 ± 1.6	<.001
E′	14.4 ± 1.9	18.1 ± 4.9	22.8 ± 5.6	11.7 ± 4.0	<.001
A′	6.4 ± 2.2	10.2 ± 2.8	16.5 ± 2.9	6.8 ± 2.7	<.001
*Inferobasal*					
S′	6.8 ± 3.0	15.0 ± 3.0	19.7 ± 4.1	9.3 ± 2.3	<.001
E′	12.0 ± 4.5	15.8 ± 4.2	20.3 ± 5.8	11.5 ± 3.6	<.001
A′	4.5 ± 2.1	9.6 ± 2.1	15.4 ± 2.7	7.0 ± 2.9	<.001

All velocities are in cm·sec^−1^; S′: peak systolic velocity; E′: peak early diastolic velocity; A′: peak late diastolic velocity. *P* values are determined using repeated measures ANOVA for within subject difference with Greenhouse-Geisser correction for the comparison of values from baseline to recovery.

**Table 4:** Interobserver variability.

	Mean	Mean difference	Limits of agreement (±2SD) (%)
LV EDV (mL)	84.5	7.2	4.5 (5.3)
LV ESV (mL)	40.5	4.3	2.4 (6.0)
EF (%)	53.5	1.2	2.3 (4.3)
Left Atrial area (cm^2^)	14.4	0.4	0.6 (4.3)
Interventricularseptal thickness (cm)	0.8	0.0	0.1 (7.7)
Posterior wall thickness (cm)	0.8	0.1	0.0 (5.5)
LV internal dimension (diastole) (cm)	4.3	0.4	0.2 (4.2)
LV mass (g)	119.4	7.3	16.3 (13.7)
*Tissue Doppler imaging velocities*			
S′	8.3	0.3	0.3 (3.1)
E′	12.8	0.1	0.2 (1.4)
A′	5.3	0.2	0.1 (2.1)

All velocities are in cm·s^−1^; S′: peak systolic velocity; E′: peak early diastolic velocity; A′: peak late diastolic velocity; mean: average of two observers; Mean difference: difference of means between two observers expressed as a positive value. Limits of agreement: two standard deviations of the difference as a percentage of the average value of the mean difference. Percentage (%) is relative to the mean.
